# Distinction Between Capping Agent and Self‐trapped Exciton in Emission Spectra

**DOI:** 10.1002/advs.78653

**Published:** 2026-11-09

**Authors:** Peter A. Tanner, Ka‐Leung Wong

**Affiliations:** ^1^ Department of Chemistry The Hong Kong Polytechnic University Kowloon Hong Kong SAR People's Republic of China; ^2^ Department of Chemistry City University of Hong Kong Kowloon Hong Kong SAR People's Republic of China

In low‐temperature studies of alkali halides published 50 or so years ago, some broad emission bands were fundamentally assigned to self‐trapped excitons (STEs) [1, 2, 3]. A STE is essentially a localized exciton featuring an emission peak maximum heavily red‐shifted relative to the free exciton emission. In these systems, strong electron‐phonon coupling distorts the lattice and traps the exciton, preventing it from traveling freely through the lattice. The classic experimental characteristics of a STE include (i) a broad emission band with wide full width at half maximum (FWHM); (ii) a substantial Stokes shift; (iii) a microsecond‐range emission lifetime; (iv) strong thermal quenching at room temperature dominated by nonradiative pathways. For example, the triplet STE emission in NaCl is located at the ultraviolet‐visible boundary at ∼360 nm (3.4 eV) with a FWHM of 0.67 eV and a lifetime of several hundred µs, but it undergoes aggressive quenching above 70 K [1, 4, 5].

It is therefore unexpected to learn that a room temperature STE was recently reported in Cs_2_NaYb_0.94_Er_0.06_Cl_6_ nanoparticles under 373 nm excitation (Figure 2b in ref. [6], redrawn here in part from digitized data, in Figure 1a, black). In that reported spectrum, the emission band maximum occurs at ∼440 nm, whereas the corresponding photoluminescence excitation (PLE) band is near 370 nm. Crucially, the ion Yb^3+^ in Cs_2_NaYbCl_6_ lacks energy levels near 370 nm [7], and while a broad absorption band for Er^3+^ in Cs_2_NaErCl_6_ exists near 380 nm [8], broad room‐temperature emission near 440 nm is absent for Cs_2_NaYbCl_6_ [7], Cs_2_NaErCl_6_ [9] or the co‐doped system Cs_2_NaYb_x_Er_1‐x_Cl_6_ [10]. Furthermore, although the emission band in ref. [6] is quite broad, the Stokes shift of ∼60 nm is not large, and the reported lifetime of between 8–10 ns (Figure S15 in [6]) is orders of magnitude faster than expected for a STE. An alternative origin for this emission band must therefore be evaluated.

**FIGURE 1:**
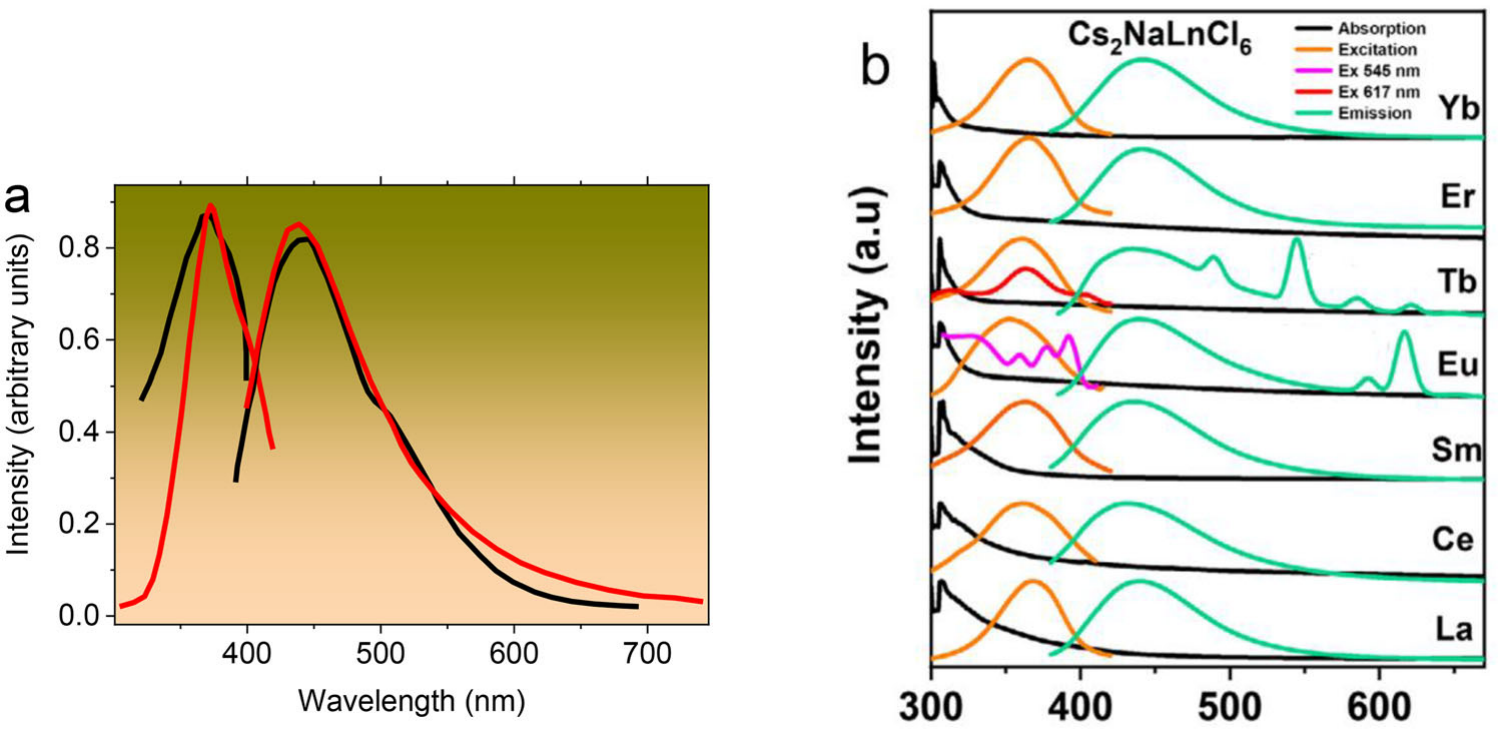
(a) Black curves: 300 K excitation (λ_em_ = 445 nm) and emission (λ_exc_ = 373 nm) spectra redrawn from digitized data from part of Figure 2b of ref. [6], originally attributed to Cs_2_NaYb_0.94_Er_0.06_Cl_6_. Red curves: Room temperature excitation (λ_em_ = 441 nm) and emission (λ_exc_ = 371 nm) spectra of oleic acid in liquid form. (Redrawn from digitized data from Ref. [11]). (b) Absorption, emission and excitation spectra of the Cs_2_NaLnCl_6_ series showing identical profiles (reprinted with permission from Ref. 17).

Because colloidal nanoparticles are highly reactive, their synthesis routinely requires surface‐passivating organic agents. The synthesis protocol in Ref. [6] utilized oleic acid (C_17_H_33_COOH) as a capping agent to bind polar carboxyl groups to the nanoparticle surface to prevent agglomeration, alongside oleylamine (C_18_H_35_NH_2_) as a reducing agent. When combined, these reagents readily form an acid–base ion pair complex that optimizes reduction kinetics and surface passivation, yielding highly monodisperse nanostructures [12].

Notably, the room‐temperature PLE and emission spectra of commercial oleic acid reported by Sekar et al. [11] (displayed in red from digitized data in Figure 1a) closely mirror the profiles attributed to the elpasolite nanoparticles in ref. [6]. The established luminescence lifetime of “oleic acid” is <10 ns [13, 14, 15], aligning with the nanosecond dynamics under dispute. While pure oleylamine lacks a prominent intrinsic photoluminescence or fluorescence emission spectrum under normal conditions, it can exhibit blue emission when forming nanoscale molecular clusters or interacting in binary solvent systems [16].

Based on these identical optical profiles and lifetime dynamics, we reassign the ∼440 nm emission band originally attributed to a STE in Cs_2_NaYb_0.94_Er_0.06_Cl_6_ [6] to the emission of the surface‐adsorbed “oleic acid” capping agent. Although the authors of ref. [6] have argued in their supplementary information (Figure S17) that the emission wavelengths and luminescence lifetimes of oleic acid differ from those of their nanoparticles, these minor differences (∼10 nm shifts and minor nanosecond variations) are easily explained by the background superposition of other compounds and/or decomposition products of oleic acid and the physical constraints of surface‐adsorbed versus free‐liquid ligands.

This reassignment is further substantiated by three key physical facts:

- **Energy Level Incompatibility**: There are no available electronic states in Cs_2_NaYb_0.94_Er_0.06_Cl_6_ capable of generating a STE under ∼370 nm excitation, as the fundamental band gaps lie at significantly shorter wavelengths [7, 8, 9]. Assumed STE mechanisms require alignment with host energy bands.
- **Scale Dependence**: This emission band is completely absent in micron‐sized, bulk Cs_2_NaYb_1‐x_Er_x_Cl_6_ [10]. prepared without organic surfactants, isolating the phenomenon to the nanoparticle synthesis chemistry.
- **Kinetics**: The nanosecond decay kinetics match organic ligand fluorescence and are at least three orders of magnitude too fast for a traditional triplet STE [13, 14, 15].

Widespread evidence of this misassignment can be found across recent literature.

For instance, Sun et al. [17] (Figure 1b) reported nearly identical excitation (∼365 nm) and emission (∼441 nm) bands for an entire series of Cs_2_NaLnCl_6_ nanoparticles (Figure 1b: Ln = Yb, Er, Tb, Eu, Sm, Ce, La). Furthermore, they observed virtually identical profiles (band shifts of <10 nm) when varying the halide in Cs_2_NaLnX_6_ (X = Cl, Br, I), assigning the bands uniformly to STEs. We consider that it is not possible for STEs of such diverse materials to have wavelengths that are so similar. For comparison, true triplet STEs in sodium halides shift drastically based on the anion: NaCl ∼360 nm, NaBr ∼270 nm, and NaI ∼295 nm. Besides the different band gaps, these differences are driven by three interconnected physical properties: anion electronegativity, lattice relaxation, and the anion atomic radius.

The assignment of STE states to Cs_2_NaLnCl_6_ nanoparticle systems has been widely promulgated in recent literature [18, 19, 20, 21, 22] and summarized uncritically in reviews [23]. Rectifying this assignment is essential to halt the propagation of incorrect physical models in halide perovskite derivatives.

Researchers should note an additional complication: even in Cs_2_NaLnCl_6_ micron‐sized particles prepared by hydrothermal routes without organic ligands, an emission band can appear near 450–470 nm [24, 25, 26, 27]. This alternative emission is due to traces of Sb^3+^ impurity and may be recognized by the characteristic PLE signature of the antimony activator.

The use of oleic acid and oleylamine is widespread for other nanoparticles such as CsPbBr_3_ [28], NaYF_4_ [29], ZnO [30] and Y_2_O_3_ [11]. Figure 2 shows the control experiment for the emission of Y_2_O_3_:Eu^3+^ with and without the addition of the capping agents. The ^5^D_0_ → ^7^F*_J_* (*J* = 0, 1, 2) emission (bottom figure) is accompanied by the emission of “oleic acid” (top figure).

**FIGURE 2:**
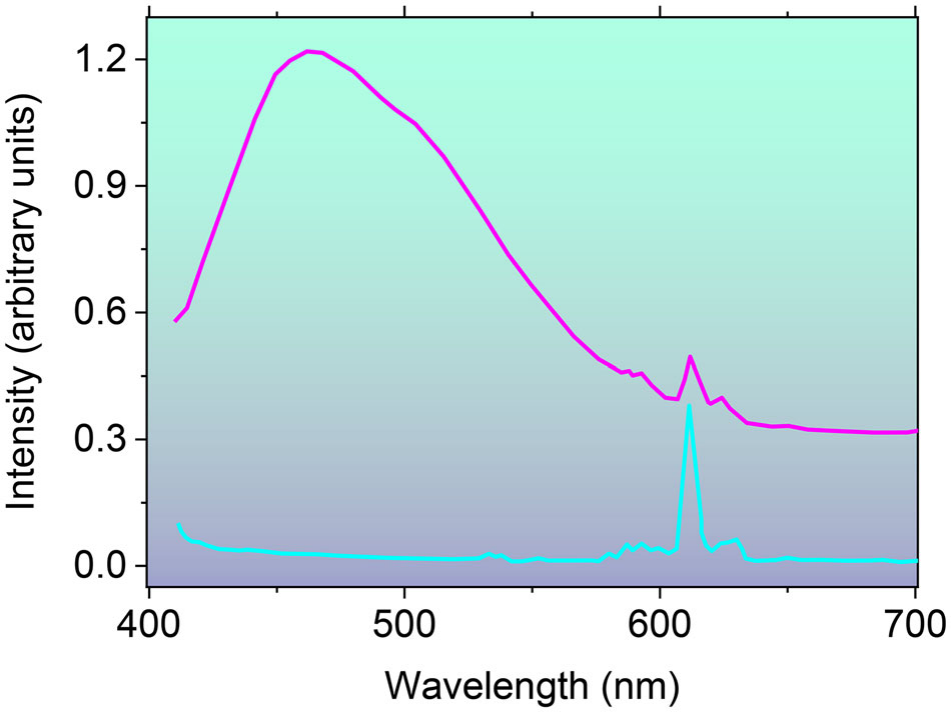
394 nm excited emission spectra of Y_1.99_Eu_0.01_O_3_ (bottom) and Y_1.99_Eu_0.01_O_3_ capped with oleic acid (top). The relative intensities are arbitrary. (Redrawn from digitized data from Figure S9 in ref. [11]).

The binding modes of oleic acid in nanoparticles have been classified using nuclear magnetic resonance and Fourier‐transform infrared spectrometry [30, 31]. In NaYF_4_, oleate binds both to Na^+^ and Y^3+^ [30]. In ZnAl_2_O_4_:Eu^3+^, the coordination to surface atoms was found to change from bridging bidentate to chelating bidentate with increasing Eu^3+^ concentration [11]. Weakly bound oleic acid coexists with PbS quantum dots [32].

Both oleic acid and oleylamine comprise cis‐ and trans‐ isomers. As‐purchased oleic acid is impure, and the effect of recrystallization moves the absorption band further into the shorter wavelength region, toward 210 nm [33], whereas impure oleylamine gives turquoise luminescence [34]. Oleic acid only comprises two chromophores: >C = C< and COO^−^. The π → π* absorption maximum λ_max_ is at ∼170–175 nm for isolated/simple alkenes. The transition types n → π* and π → π* have the absorption maximum at ∼190–215 nm for isolated carboxylate groups or simple carboxylate ions. The lifetime of the “oleate” emission is a few ns, and the excitation band has λ_max_ at ∼370 nm, so that the excited state is a singlet. First, from the different excitation spectra of the peak maximum and the shoulder [11], there are several emissive species. Second, the oxidation of oleic acid leads to the formation of hydroperoxides, conjugated polyenes, and secondary products like α,β‐unsaturated carbonyls. These species are fluorescent, for example in the 440 nm range, as shown by the emission spectra of corn oil [35] or olive oil [36]. Hence, although oleic acid is the capping agent, the emission derives from oxidized species.

## Conflicts of Interest

The authors declare no conflicts of interest.

## Data Availability

The data in this work will be made available upon reasonable request to the authors.
